# Physical activity and vascular disease in a prospective cohort study of older men: The Health In Men Study (HIMS)

**DOI:** 10.1186/s12877-015-0157-2

**Published:** 2015-12-09

**Authors:** Ben Lacey, Jonathan Golledge, Bu B. Yeap, Sarah Lewington, Paul E. Norman, Leon Flicker, Osvaldo P. Almeida, Graeme J. Hankey

**Affiliations:** Western Australian Centre for Health & Ageing, University of Western Australia, Crawley, Australia; School of Medicine and Pharmacology, University of Western Australia, Crawley, Australia; Queensland Research Centre for Peripheral Vascular Disease, College of Medicine and Dentistry, James Cook University, Townsville, Australia; The Department of Vascular and Endovascular Surgery, The Townsville Hospital, Townsville, Australia; Department of Endocrinology and Diabetes, Fiona Stanley and Fremantle Hospitals, Perth, Australia; Clinical Trial Service Unit and Epidemiological Studies Unit (CTSU), Nuffield Department of Population Health, University of Oxford, Oxford, United Kingdom; School of Surgery, University of Western Australia, Crawley, Australia; Department of Geriatric Medicine, Royal Perth Hospital, Perth, Australia; School of Psychiatry and Clinical Neurosciences, University of Western Australia, Crawley, Australia; Department of Neurology, Sir Charles Gairdner Hospital, Perth, Australia; WA Centre for Health & Ageing (M573), University of Western Australia, 35 Stirling Highway, Crawley, WA 6009 Australia

**Keywords:** Epidemiology, Cardiovascular diseases, Exercise

## Abstract

**Background:**

The dose–response relationship between volume of physical activity and incidence of major vascular events at older age is unclear. We aimed to investigate this association in a cohort of older men.

**Methods:**

For this prospective cohort study, 7564 men aged 65–83 years and without prior vascular disease were recruited in 1996–99 from the general population in Perth, Western Australia. Men were followed up using the Western Australian Data Linkage System to identify deaths and hospitalisations. During mean follow-up of 11 (SD 4) years, there were 1557 first major vascular events: 833 ischaemic heart disease events, 551 stroke events and 173 other vascular events. Cox regression was used to calculate hazard ratios (adjusted for age, education and smoking) for incidence of major vascular events by volume of baseline recreational physical activity (measured in metabolic equivalent [MET] hours per week).

**Results:**

Hazard ratios among men who performed 0, 1–14, 15–24, 25–39, ≥40 MET-hours per week of recreational physical activity were 1.00 (95 % CI 0.91–1.10; referent), 0.88 (0.79–1.00), 0.81 (0.72–0.91), 0.81 (0.72–0.91) and 0.80 (0.71–0.89), respectively (P _trend_ =0.006). The association was slightly attenuated with further adjustment for BMI. There was evidence of stronger associations at older ages and greater intensity of activity, but no evidence of effect modification by smoking, alcohol intake or BMI. There was also no evidence that the association varied by type of vascular event.

**Conclusions:**

Among men aged over 65 years, there was a curvilinear association between recreational physical activity and incidence of major vascular events, with an inverse association up to about 20 MET-hours per week (equivalent to 1 h of non-vigorous, or half an hour of vigorous, physical activity per day) and no evidence of further reductions in risk thereafter.

**Electronic supplementary material:**

The online version of this article (doi:10.1186/s12877-015-0157-2) contains supplementary material, which is available to authorized users.

## Background

Physical inactivity is an established risk factor for vascular disease [1]. Despite this, levels of activity are low in many populations and increasing efforts are being made to promote exercise, especially at older ages when the absolute risk of vascular disease is high [2, 3]. The World Health Organisation recommends that those over 65 years of age should do at least 75 mins of vigorous-intensity exercise, or 150 mins of moderate-intensity exercise, per week (7.5 metabolic equivalent [MET] hours per week) [4]. Uncertainties remain, however, regarding the shape and strength of the association between volume of physical activity and vascular disease.

Volume of physical activity has been inversely associated with both ischaemic heart disease [5–8] and stroke [9–11]. The lack of consistency in how physical activity is defined and measured in different studies means there is limited evidence on the dose–response relationship between volume of physical activity and risk of major vascular events. Those few meta-analyses which have combined the results of studies using standardised measures of physical activity, such as kcal per week or MET-hours per week, describe a curvilinear dose–response relationship with a strong inverse association at lower levels of physical activity that becomes progressively shallower at higher levels [6, 7, 12]. In these studies, high levels of physical activity are association with ~20–40 % lower risk of major vascular events relative to those with very low levels of physical activity. A similar curvilinear relationship has been described in studies of the association between physical activity and all-cause mortality [12–14].

Meta-analyses which describe the relationship between physical activity and major vascular events have been of mainly middle-aged adults and it remains unclear whether the association differs at older age [7]. It also remains unclear whether the association varies by type of major vascular event (such as ischaemic heart disease and stroke), by intensity of physical activity performed or by other lifestyle factors, such as smoking, alcohol intake and adiposity. Furthermore, there is some evidence from recent studies that vascular risk may increase at very high levels of physical activity, and this has not been fully assessed at older age [15, 16].

We report the findings from a population-based prospective cohort study of older men in Western Australia. The main aims of this report were: 1) to quantify the dose–response association between volume of recreational physical activity and incidence of major vascular events at older age; 2) to investigate whether the association varied by type of major vascular event; and, 3) to assess whether age, other lifestyle factors (smoking, alcohol intake or BMI) or intensity of activity modified the association.

## Methods

### Study design and participants

For this prospective cohort study, 12 203 men aged 65–83 years were recruited in 1996–99 from the general population in Perth, Western Australia. These men were initially part of a larger population-based randomised trial of screening for abdominal aortic aneurysm. The trial methods have been described in detail elsewhere [17]. In brief, men were randomly selected from the general population in Perth to undergo ultrasound screening of their abdominal aorta. Those screened received a letter (together with a copy for their general practitioner) reporting the size of their aorta, but no attempt was made to influence the clinical management of the general practitioner and no further intervention was given as part of the trial. At screening, the men also completed a questionnaire, which included an assessment of physical activity performed in a usual week.

Outcomes in this cohort were monitored by the Western Australian Data Linkage System [18], which has records of deaths and hospital discharge diagnoses in Western Australia from 1970 onwards. The system allowed the underlying cause of death and the discharge diagnoses for inpatient hospital admissions throughout Western Australia to be obtained, coded to 3 digits using ICD-10.

For this report, we excluded men identified as having an enlarged abdominal aorta (≥30 mm in diameter) at screening (*n* = 875), as they are likely to have received medical intervention during follow-up to address their risk factors for vascular disease. We also excluded those with a past history of heart disease (*n* = 3419) or stroke (*n* = 1735), to limit the effect of reverse causality (past medical history was identified from both self-reported disease at baseline and the historical records of the Western Australian Data Linkage System). We further excluded men with missing data from the questionnaire on key variables (physical activity, age, education and BMI; *n* = 54). The remaining 7564 men contributed person-years until first major vascular event, death or the censoring date (Dec 31, 2010).

Ethics approval for the study was obtained from the Human Research Ethic Committee of the University of Western Australia, and all men provided written informed consent to participate.

### Procedures

The questionnaire, completed at the same time as aortic screening, provided information on socio-demographic factors, medical history and lifestyle. Physical measurements were also made, including height (to 0.5 cm) and weight (to 0.2 Kg). To assess physical activity, men were asked if they currently undertook recreational exercise in a usual week and, if so, the time (hours and minutes) spent performing ‘non-vigorous’ and ‘vigorous’ exercise, separately. Examples of each type of activity were given: slow walking, slow cycling, Tai Chi and Yoga for non-vigorous activity; and fast walking, jogging, aerobics, vigorous swimming, vigorous cycling, tennis, football and squash for vigorous activity.

Total MET-hours per week were estimated for each participant. MET values quantify energy expenditure relative to an individual’s rate of energy expenditure at rest (their resting metabolic rate). Non-vigorous activity was assigned a MET value of three and vigorous activity a MET value of five, in line with the suggested values of such activities for people aged 65–79 years in the US Surgeon General’s Report on Physical Activity [19]. Total MET-hours per week were calculated, therefore, as the sum of the hours of non-vigorous activity multiplied by three and the hours of vigorous activity multiplied by five. For the main analyses, active men were divided into quartiles of MET-hours per week which, together with inactive men (those performing 0 MET-hours per week), produced five groups: 0, 1–14, 15–24, 25–39, ≥40 MET-hours per week.

The primary outcome for the analyses was first major vascular event. This was a composite endpoint of ischaemic heart disease (non-fatal myocardial infarction [ICD-10: I21-23] or ischaemic heart disease death [I20-25]), stroke (non-fatal stroke or stroke death [I60-61, I63-64, H34.1]) and other vascular death (all vascular deaths [I60-99] except stroke or ischaemic heart disease) (see Additional file 1: Table S1).

### Statistical analysis

We used Cox regression (with attained age as the underlying time variable) to calculate hazard ratios for first major vascular event by level of MET-hours per week, with inactive men as the reference group. In addition to age at risk (ie, age during follow-up), analyses were adjusted for education (some primary, some high school, completed high school, completed university) and smoking (never smoker, ex-smoker, current smoker), selected *a priori* as important confounders. Assessment was made for further confounding by adjusting for region of birth (Australia, Europe, Mediterranean, other), quantity of weekly alcohol intake (none, 1–7, 8–14, ≥15 units), marital status (never married, previously married, currently married), frequency that salt was added to food (rarely/never, sometimes, always/almost always), self-reported diabetes (yes, no), use of blood pressure medication (yes, no) and use of cholesterol medication (yes, no). Missing values formed a separate category in each variable; information on alcohol intake, frequency that salt was added to food and medication use was not collected in 332 men. The main analyses were presented with and without adjustment for BMI (<22.5, 22.5–24.9, 25.0–27.4, 27.5–29.9, ≥ 30.0 kg/m [2]), as adiposity can be considered both a confounder and possible mediator of the effect of physical activity on vascular risk. We excluded the first 2 years of follow-up to assess for reverse causality from preclinical disease at baseline.

We calculated hazard ratios (adjusted for age at risk, education and smoking) for each type of major vascular event (ischaemic heart disease, stroke and other vascular) by level of MET-hours per week. As for the main analyses, these results were presented with and without further adjustment for BMI. Effect modification of the association for first major vascular event in active versus inactive men was assessed by stratifying on age at risk, smoking, alcohol intake, BMI and intensity of physical activity (no vigorous, some vigorous, only vigorous); the latter divided active men in half to control for variation in median MET-hours per week between strata of intensity of physical activity. Chi-squared tests for heterogeneity and trend, when appropriate, were applied to hazard ratios for each of these variables.

The 95 % confidence intervals about the hazard ratios were calculated using the variance of the log risk [20]. This appropriately attributes variance to the reference, and other, groups to allow confidence intervals to be used to compare risks in any two groups, rather than solely between the reference group and another group. The absolute risks by level of physical activity were examined by multiplying hazard ratios by a common factor to make the inverse-variance weighted average of the hazard ratios match the annual incidence of major vascular events in this cohort. [21, 22] Annual incidence rates were calculated as the unweighted average of the component 5-year incidence rates (Additional file 2: Table S2). The proportionality assumption of the Cox models was tested using Schoenfeld residuals and was found to be valid for all of the analyses. Analyses were performed using Stata (v12.0) and figures were plotted using R (v3.0).

## Results

In total, 7564 men with no previous vascular disease at baseline were included in this analysis. Mean age at recruitment was 72 years (SD 4) and they were followed for a mean of 11 (SD 4) years to Dec 31, 2010. 1557 (21 %) men had a major vascular event during follow-up, at a mean age of 80 (SD 6) years. At baseline, 5688 (75 %) men reported some recreational physical activity in a usual week. Among active men, the median physical activity was 25 (IQR 15–40) MET-hours per week (the distribution of MET-hours per week was right skewed) with 65 % (*n* = 3671) performing non-vigorous activity only, 13 % (*n* = 727) vigorous activity only and 23 % (*n* = 1290) both non-vigorous and vigorous activity.

Several vascular risk factors were associated with recreational physical activity at baseline (Table 1). In particular, physically active men were more likely than inactive men to have been educated beyond primary school, and were less likely to be current smokers and add salt to their food. A slightly higher proportion of physically active men were born in Australia and were weekly drinkers. There was also a negative association between physical activity and BMI (P _trend_ <0.0001).

**Table 1:** Characteristics of the 7564 participants, by recreational physical activity at baseline

Characteristics	Recreational physical activity		
	0 MET-hours per week	1–24 MET-hours per week	≥25 MET-hours per week
Number of participants, n	1876	2812	2876
Mean (SD) age, years	71.9 (4.4)	71.5 (4.3)	71.6 (4.3)
No education beyond primary school, n (%)	533 (28.4)	571 (20.3)	511 (17.8)
Born in Australia, n (%)	982 (52.3)	1507 (53.6)	1627 (56.6)
Currently married, n (%)	1510 (80.5)	2277 (81.0)	2328 (80.9)
Current smokers, n (%)	339 (18.1)	238 (8.5)	263 (9.1)
Weekly drinkers, n (%)^a^	1180 (64.7)	1829 (67.5)	1950 (72.3)
Mean (SD) BMI, kg/m^2^	27.1 (4.1)	26.9 (3.6)	26.3 (3.4)
Self-reported diabetes, n (%)	177 (9.4)	320 (11.4)	244 (8.5)
Always/almost always adds salt to food, n (%)^a^	708 (38.8)	761 (28.1)	762 (28.2)
Using blood pressure-lowering medication, n (%)^a^	487 (26.7)	794 (29.3)	705 (26.1)
Using cholesterol-lowering medication, n (%)^a^	189 (10.4)	288 (10.6)	263 (9.7)
Mean (SD) person-years of observation	10.0 (4.1)	10.6 (3.9)	10.8 (3.9)
Number of major vascular events, n (%)	443 (23.6)	556 (19.8)	558 (19.4)

Active men were divided in half (1–24, ≥25 MET-hours per week)

^a^Information on alcohol intake, frequency that salt is added to food and medication use was not collected in 332 men

**Fig. 1:**
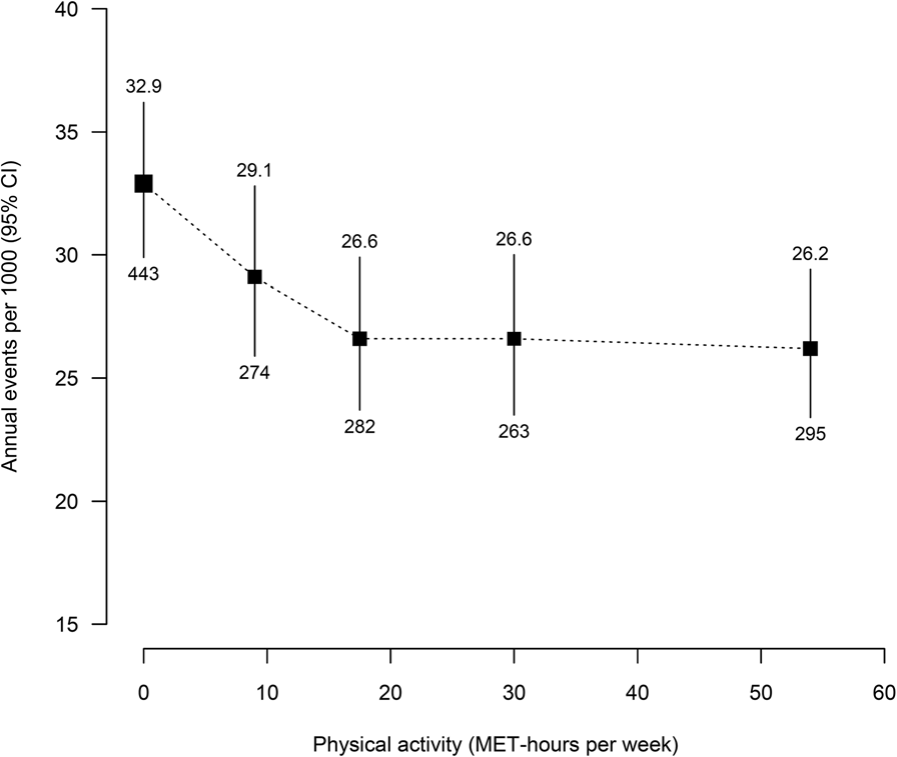
Incidence of major vascular events versus recreational physical activity (among 7564 participants). Hazard ratios (adjusted for age at risk, education and smoking) at ages 65–94 years for major vascular events versus physical activity were multiplied by a common factor (i.e. floated) to make the weighted average match the annual incidence of major vascular events in this cohort. Mean age at event was 80 years. Annual incidence was the unweighted average of the component 5-year incidence rates. Physical activity was subdivided into five prespecified categories (0, 1–14, 15–24, 25–39, ≥40 MET-hours per week) and hazard ratios plotted against the median of each category. For each category, area of square is inversely proportional to the variance of the category-specific log risk, which also determines the confidence interval. Incidence shown above each square and numbers of events below

Overall, there was a curvilinear association between recreational physical activity and incidence of major vascular events, with an inverse association up to about 20 MET-hours per week and no evidence of further reductions in risk thereafter (Table 2, Fig. 1). Hazard ratios (adjusted for age at risk, education and smoking) among men who performed 0, 1–14, 15–24, 25–39, ≥40 MET-hours per week were 1.00 (95 % CI 0.91–1.10; referent), 0.88 (0.79–1.00), 0.81 (0.72–0.91), 0.81 (0.72–0.91) and 0.80 (0.71–0.89), respectively (P _trend_ =0.006). In the lower physical activity range (<25 MET-hours per week), the association was approximately log-linear: 10 MET-hours per week greater physical activity was associated with 12 % lower risk of major vascular events (hazard ratio 0.88 [95 % CI 0.81–0.96], *P* = 0.002). Adjusting for other potential confounders did not materially alter the associations (Table 2; Additional file 3: Table S3) and neither did exclusion of the first 2 years of follow-up (Additional file 4: Table S4). Further adjustment for BMI had only a slight attenuating effect on the associations (Table 2; Additional file 5: Table S5).

**Table 2:** Hazard ratios for incidence of major vascular events versus recreational physical activity, with and without adjustment for potential confounders (among 7564 participants)

Physical activity, MET-hours per week	Median MET-hours per week	Number of events	Hazard ratio (95 % CI)		
			Adjusted for age at risk, education and smoking	Adjusted for age age at risk, education, smoking and other factors^a^	Adjusted for age age at risk, education, smoking, other factors^a^ and BMI
0	0.0	443	1.00 (0.91–1.10)	1.00 (0.91–1.10)	1.00 (0.91–1.10)
1–14	9.0	274	0.88 (0.79–1.00)	0.85 (0.75–0.96)	0.86 (0.76–0.97)
15–24	17.5	282	0.81 (0.72–0.91)	0.81 (0.72–0.91)	0.83 (0.73–0.93)
25–39	30.0	263	0.81 (0.72–0.91)	0.80 (0.71–0.91)	0.83 (0.73–0.94)
≥40	54.0	295	0.80 (0.71–0.89)	0.80 (0.71–0.91)	0.83 (0.74–0.94)
			Trend, 5 groups: χ^2^_1_ = 8.6 (*P* = 0.003)	Trend, 5 groups: χ^2^_1_ = 8.2 (*P* = 0.004)	Trend, 5 groups: χ^2^_1_ = 5.1 (*P* = 0.02)

^a^Other factors: place of birth, marital status, quantity of weekly alcohol intake, frequency that salt is added to food, use of blood pressure-lowering medication, use of cholesterol-lowering medication and diabetes

**Fig. 2:**
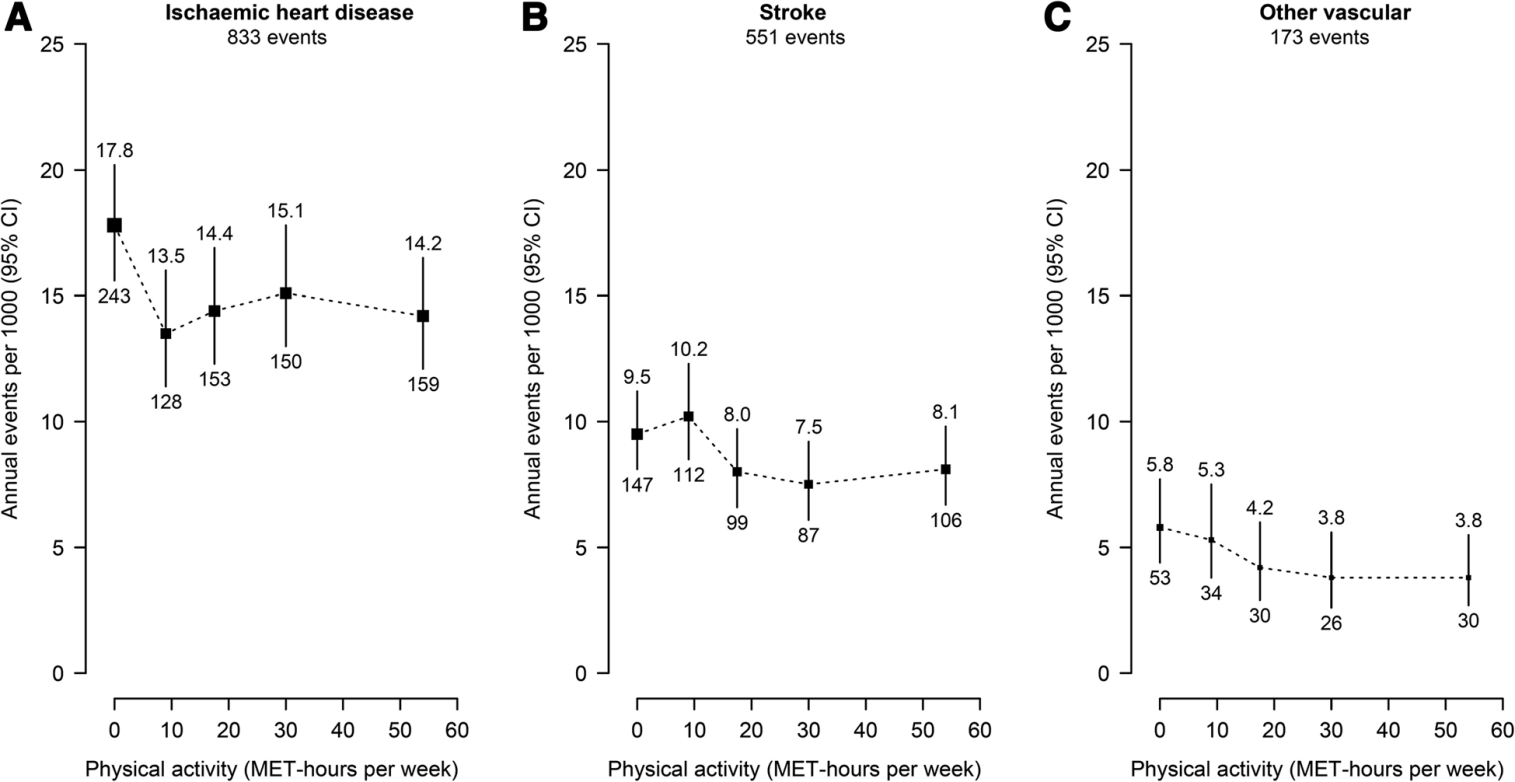
Cause-specific incidence of major vascular evens versus recreational physical activity (among 7564 participants). Hazard ratios (adjusted for age at risk, education and smoking) at ages 65–94 years for each type of vascular event versus physical activity were multiplied by a common factor (i.e. floated) to make the weighted average match the annual incidence of each type of vascular event in this cohort. Annual incidence was the unweighted average of the component 5-year incidence rates. Physical activity was subdivided into five prespecified categories (0, 1–14, 15–24, 25–39, ≥40 MET-hours per week) and hazard ratios plotted against the median of each category. For each category, area of square is inversely proportional to the variance of the category-specific log risk, which also determines the confidence interval. Incidence shown above each square and numbers of events below

Ischaemic heart disease accounted for over half of the major vascular events (*n* = 833), stroke for about a third (*n* = 551) and the remainder were other vascular deaths (*n* = 173); for further details on the type of other vascular deaths, see Additional file 2: Table S2. There was no strong evidence that the shape or strength of the association differed by type of major vascular event (Tables 3; Fig. 2). Relative to inactive men, the hazard ratios (adjusted for age at risk, education and smoking) for all active men combined were 0.81 (95 % CI 0.69–0.94) for ischaemic heart disease, 0.88 (0.73–1.07) for stroke and 0.73 (0.53–1.02) for other vascular death (P _heterogeneity_ =0.8). As with the overall association, further adjustment for BMI had only a slight attenuating effect on these associations (Additional file 5: Table S5).

**Table 3:** Hazard ratios for cause-specific incidence of major vascular events versus recreational physical activity (among 7564 participants)

Physical activity, MET-hours per week	Median MET-hours per week	Ischaemic heart disease		Stroke		Other vascular	
		n	Hazard ratio (95 % CI)	n	Hazard ratio (95 % CI)	n	Hazard ratio (95 % CI)
0	0.0	243	1.00 (0.88–1.14)	147	1.00 (0.85–1.18)	53	1.00 (0.76–1.32)
1–14	9.0	128	0.76 (0.64–0.90)	112	1.07 (0.89–1.29)	34	0.92 (0.66–1.29)
15–24	17.5	153	0.81 (0.69–0.95)	99	0.84 (0.69–1.02)	30	0.72 (0.50–1.03)
25–39	30.0	150	0.85 (0.73–1.00)	87	0.79 (0.64–0.97)	26	0.66 (0.45–0.97)
≥40	54.0	159	0.80 (0.68–0.93)	106	0.85 (0.71–1.03)	30	0.66 (0.46–0.94)
		Trend, 5 groups: χ^2^_1_ = 2.6 (*P* = 0.11)		Trend, 5 groups: χ^2^_1_ = 3.3 (*P* = 0.07)		Trend, 5 groups: χ^2^_1_ = 4.3 (*P* = 0.04)	

Hazard ratios adjusted for age at risk, education and smoking; for hazard ratios further adjusted for BMI, see Additional file 5: Table S5

For major vascular events combined, the association was found to be stronger at older ages in active men relative to inactive men (P _trend_ =0.04; Table 4). However, after exclusion of the first 2 years of follow-up, the evidence for this was less strong (P _trend_ =0.10; Additional file 4: Table S4). There was no significant heterogeneity across the associations when stratified by smoking (*P* = 0.6), alcohol intake (*P* = 1.0) or BMI (P _trend_ =0.6). When stratified by intensity of activity, there was evidence of a greater effect with more vigorous activity performed at each level of MET-hours per week (Table 5). However, even in active men who performed no vigorous activity, there continued to be evidence of a lower risk of major vascular events relative to inactive men (hazard ratio 0.85 [95 % CI 0.80–0.92]).

**Table 4:** Hazard ratios for incidence of major vascular events in active versus inactive men, by baseline variables (among 7564 participants)

	Number of events	Mean age at event, years	Hazard ratio (95 % CI)
Age at risk, years			
65–74	316	71.8	0.94 (0.75–1.34)
75–84	936	79.9	0.85 (0.81–0.91)
85–94	305	88.0	0.65 (0.51–0.83)
			Trend, 3 groups: χ^2^_1_ = 4.3 (*P* = 0.04)
Smoking			
Never	469	80.3	0.90 (0.81–1.00)
Ex-smoker	868	80.0	0.78 (0.72–0.85)
Current	220	78.1	0.83 (0.69–0.99)
			Heterogeneity: χ^2^_2_ = 2.1 (*P* = 0.15)
Alcohol intake ^a^			
≥ weekly	491	79.9	0.83 (0.74–0.92)
< weekly	990	79.8	0.83 (0.77–0.89)
			Heterogeneity: χ^2^_1_ = 0.0 (*P* = 1.0)
BMI, kg/m^2^			
14–24	463	80.5	0.88 (0.79–0.97)
25–29	755	79.6	0.85 (0.78–0.92)
30–47	339	79.3	0.81 (0.70–0.92)
			Trend, 3 groups: χ^2^_1_ = 0.9 (*P* = 0.3)
Overall	1557	79.8	0.82 (0.78–0.87)

Hazard ratios were adjusted for age at risk, education and smoking

^a^Information on alcohol intake was not collected in 332 men

**Table 5:** Hazard ratios for incidence of major vascular events, by volume and intensity of recreational physical activity (among 7564 participants)

Physical activity	Number of events	Mean age at event, years	Hazard ratio (95 % CI)
1–24 MET-hours per week			
No vigorous	360	79.8	0.86 (0.77–0.95)
Some vigorous	80	80.1	0.83 (0.67–1.03)
Only vigorous	116	79.0	0.81 (0.67–0.97)
			Trend, 3 groups: χ^2^_1_ = 0.3 (*P* = 0.6)
≥25 MET-hours per week			
No vigorous	398	79.8	0.85 (0.77–0.93)
Some vigorous	145	79.2	0.71 (0.60–0.84)
Only vigorous	15	81.1	0.64 (0.39–1.06)
			Trend, 3 groups: χ^2^_1_ = 4.1 (*P* = 0.04)

Hazard ratios were adjusted for age at risk, education and smoking. Participants reporting no recreational physical activity were referent

## Discussion

In this prospective cohort study of 7564 men, aged over 65 years and without vascular disease at baseline, there was an inverse association between volume of recreational physical activity and incidence of major vascular events up to about 20 MET-hours per week (associated with about 20 % lower risk of major vascular events relative to inactive men) and no evidence of further reductions in risk thereafter. There was evidence of stronger associations at older ages and greater intensity of activity, but no evidence of effect modification by smoking, alcohol intake or BMI. Neither was there strong evidence that the association varied by type of major vascular event.

These findings are broadly consistent with other large prospective cohort studies, which have assessed this relationship in mainly middle-aged adults. A recent meta-analysis reported a 23 % (95 % CI 18–30 %) reduction in major vascular events in men who performed high, versus low, levels of recreational physical activity, with a 21 % (15–27 %) reduction in ischaemic heart disease and a 29 %(16–40 %) reduction for stroke [11].

Few meta-analyses have assessed the dose–response relationship between physical activity and major vascular events. One meta-analysis which described the dose–response relationship between recreational physical activity and ischaemic heart disease in men, reported an association similar in shape and strength to the overall association in the present study: relative to inactive men, 11 MET-hours per week of recreational physical activity was associated with 9 % (95 % CI −5–21 %) lower risk and 22 MET-hours per week was associated with 18 % (9–26 %) lower risk; higher volumes of activity were associated with only slight further reductions in risk [6]. Another meta-analysis of the dose–response relationship described a similar curvilinear association but with somewhat greater relative risks: there was inverse association up to 15.0–22.5 MET-hours per week of recreational physical activity which was associated with 41 % (95 % CI 37–43 %) lower risk of death from heart disease relative to inactive adults [12]. The sex-specific findings for this association were not reported, although the association with all-cause mortality was noted to be shallower in men than women. Studies consistently describe an inverse association between volume of physical activity and stroke, but the shape and strength of the dose–response relationship has not been well described [9, 11].

None of the identified meta-analyses describe the association between physical activity and vascular events by age. A systematic review of the relationship between physical activity and cardiovascular disease reported that in individual studies of older men and women, the risk reduction comparing the most active to the least active adults were similar in magnitude to studies of younger adults, although there was substantial uncertainty about the estimates at older age [7]. The Harvard Alumni Health Study (one of the largest studies of the effects of physical activity in older men, with mean age at entry 66 years) reported a curvilinear association between total physical activity and risk of coronary heart disease: relative to men who performed <4200 kJ per week of physical activity per week and adjusting for age, those who performed 4200–8399 kJ per week had a 15 % (95 % CI 3–41 %) lower risk of coronary heart disease and those in the highest physical activity group (≥16 800 kJ per week) had 30 % (9–47 %) lower risk [23].

There is not a clear reason for the effect modification by age described in the present study. The proportion of men performing at least some vigorous activity fell with increasing age in the present study, indicating that intensity of activity is unlikely to account for this variation. Reverse causality is a possible explanation, despite measures to limit its effect, particularly given the weaker evidence following exclusion of the first 2 years of follow-up. However, these results may still represent a greater effect of physical activity with increasing age.

There is no strong evidence in the literature to suggest that smoking, alcohol intake or BMI modify the strength of the association between physical activity and vascular risk, consistent with present study [24]. There is some evidence from other studies that vigorous exercise may have an effect on ischaemic heart disease beyond its contribution to MET-hours per week and the findings of the present study support this [8, 25].

This study had a number of key strengths. The extensive baseline survey allowed assessment for confounding by a number of socio-demographic and lifestyle factors, although a greater range of dietary variables would have been ideal. The study also benefited from an established data linkage system to identify the causes of deaths and hospitalisations in study participants; the system also allowed identification of men with a past medical history of vascular disease at baseline. Case-ascertainment of incident major vascular events was likely to have been high as the population is stable and the data linkage system received information from mortality records and all hospitals throughout Western Australia [26, 27].

The study also had a number of limitations. Men were recruited from a trial of the effect of screening for abdominal aortic aneurysm and, although no treatment or clinical management was advocated as part of the trial, it is possible that men may have been encouraged (particularly those with an abdominal aortic aneurysm) to address their risk factors for vascular disease. We excluded those identified as having an abdominal aortic aneurysm at baseline to limit this effect.

Another limitation was the estimation of MET-hours per week. Quantifying physical activity, as described, has been demonstrated to be valid in a small sample of community-dwelling older adults [28], but ideally the measure would have been supplemented by more objective assessments of activity, such as information captured by an accelerometer. It is also a limitation of the measurement of physical activity that it was only assessed at recruitment, which meant it was not possible to investigate changes in physical activity over time or correct for regression dilution bias (the bias that can occur when risk is plotted against baseline levels of an exposure as opposed to long-term average levels) [29].

At baseline, participants were asked solely about recreational forms of physical activity and, as such, this study was not able to assess the effects of total volume of physical activity on risk of major vascular events (participants did not report physical activity for transport, domestic or occupational purposes). Volume of recreational physical activity is likely to be correlated with total volume of physical activity, but the nature of this relationship and its variation with age is unclear. This study was also not able to assess: the independent effect of non-vigorous and vigorous activity on risk of each type of major vascular event; the effect of frequency of exercise during a usual week; the effect of sedentary behaviors; or the strength of associations by subtype of stroke, all of which should be explored in future research.

## Conclusions

This study adds to the limited evidence on the effects of physical activity on risk of major vascular events at older age. There was an inverse association between recreational physical activity and incidence of major vascular events up to about 20 MET-hours per week (equivalent to approximately 1 h of non-vigorous, or half an hour of vigorous, physical activity per day), and no evidence of further reductions in risk thereafter. Reassuringly, there was no evidence of an increase in risk at higher levels of physical activity, even at seven times the World Health Organisation minimum physical activity recommendation. These findings supports the public health advice that men should remain active at older age, as even modest levels of non-vigorous activity appear to be of substantial benefit to vascular health.

## Additional files

Additional file 1: Table S1. Major vascular events endpoints and their ICD-9 and ICD-10 codes (PDF 5 kb) Additional file 2: Table S2. Number of major vascular events, by age at risk and type of vascular event (among 7564 participants) (PDF 73 kb) Additional file 3: Table S3. Incidence of major vascular events versus recreational physical activity, with progressive adjustment for potential confounders (among 7564 participants) (PDF 21 kb) Additional file 4: Table S4. Excluding first 2 years of follow-up: hazard ratios for incidence of major vascular events in active versus inactive men, by age at risk (among 7350 participants) (PDF 14 kb) Additional file 5: Table S5. Hazard ratios for cause-specific incidence of major vascular events versus recreational physical activity, with further adjustment for BMI (among 7564 participants) (PDF 33 kb)

## Abbreviations

MET-hours: Metabolic equivalent hours
BMI: Body mass index
HR: Hazard ratios
CI: Confidence interval
SD: Standard deviation
IQR: Interquartile range
ICD-10: International Classification of Diseases 10th revision
