# Supplementary material for: Physical activity and vascular disease in a prospective cohort study of older men: The Health In Men Study (HIMS)
Source: BMC Geriatr. 2015 Dec 9;15:164. doi: 10.1186/s12877-015-0157-2 (PMC4674929; doi:10.1186/s12877-015-0157-2)
Supplement: Additional file 1: Table S1. — Major vascular events endpoints and their ICD-9 and ICD-10 codes (PDF 5 kb) [file 12877_2015_157_MOESM1_ESM.pdf]

Supplementary Table 1: Major vascular events endpoints and their ICD-9 and ICD-10 codes

	ICD-9	ICD-10	Note
Ischaemic heart disease			
Myocardial infarction	410	I21-I23	
Other ischaemic heart disease*	411-414	I20, I24-I25	
Stroke			
Ischaemic stroke	433-434, 362.3	I63, H34.1	Includes central retinal artery occlusion
Intracerebral hemorrhage	431	I61	
Subarachnoid hemorrhage	430	I60	
Unspecified stroke	436	I64	
Other vascular disease*			
Aortic aneurysm	441	I71	
Pulmonary embolism	415	I26	
Heart failure	428	I50	
Hypertensive disease	401-405	I10-I15	Includes hypertension, hypertensive heart disease and hypertensive renal disease
Atherosclerosis & other arterial disease	440, 442-448	I70, I72-I79	Includes peripheral arterial disease, and diseases of arterioles/capillaries
Inflammatory heart disease	420-424	I30-I41	Includes pericarditis, myocarditis, endocarditis
Rheumatic heart disease	390-398	I00-I09	Includes acute and chronic rheumatic heart disease
Other heart disease (not IHD)	416-417,425-427, 429	I27-I28, I42-I49, I51-I52	Includes pulmonary heart disease, cardiomyopathy, dysrhythmia
Other cerebrovascular disease (not stroke)	435, 437-438	I62, I65-69	Includes remainder of cerebrovascular disease ICD-9/10 subchapters not classified as stroke†
Other circulatory disease	451-459	I80-I99	Includes venous disease (including oesophageal varices) and lymphatic disease
All vascular disease	390-459, 362.3	I00-99, H34.1	

* Deaths only (where disease was considered the underlying cause)
† There were no transient ischaemic attack deaths (435 or G45)

ICD-9 codes used for baseline exclusions: participants with a baseline history of major heart disease or stroke/TIA with the following ICD-9 codes: chronic rheumatic heart disease (393-398); hypertensive heart disease (402,404); pulmonary heart disease (415-416); heart failure (428); ischaemic heart disease (410-414); and stroke/TIA (362.3,430-431,433-436).
