# Supplementary material for: Physical activity and vascular disease in a prospective cohort study of older men: The Health In Men Study (HIMS)
Source: BMC Geriatr. 2015 Dec 9;15:164. doi: 10.1186/s12877-015-0157-2 (PMC4674929; doi:10.1186/s12877-015-0157-2)
Supplement: Additional file 2: Table S2. — Number of major vascular events, by age at risk and type of vascular event (among 7564 participants) (PDF 73 kb) [file 12877_2015_157_MOESM2_ESM.pdf]

Supplementary Table 2: Number of major vascular events, by age at risk and type of vascular event (among 7564 participants)

Age at risk, years	Person-years at risk	Number of events (rate per 1000 person-years)							
		Ischaemic heart disease		Stroke		Other vascular*		All	
65-69	7255	45	(6.2)	25	(3.4)	1	(0.1)	71	(9.8)
70-74	21520	155	(7.2)	78	(3.6)	12	(0.6)	245	(11.4)
75-79	27110	264	(9.7)	187	(6.9)	38	(1.4)	489	(18.0)
80-84	17169	208	(12.1)	175	(10.2)	64	(3.7)	447	(26.0)
85-89	5706	131	(23.0)	72	(12.6)	45	(7.9)	248	(43.5)
90-94	909	30	(33.0)	14	(15.4)	13	(14.3)	57	(62.7)
All ages†	79669	833	(15.2)	551	(8.7)	173	(4.7)	1557	(28.6)

* Other vascular deaths: 18 aortic aneurysm; 5 pulmonary embolism; 17 heart failure; 30 hypertensive disease; 15 atherosclerosis and other arterial disease; 20 inflammatory heart disease; 1 rheumatic heart disease; 39 other heart disease (not IHD); 21 other cerebrovascular disease (not stroke); 7 other circulatory disease.

† Age-standardised rates by taking the unweighted average of the component five-year rates.
