# Supplementary material for: Physical activity and vascular disease in a prospective cohort study of older men: The Health In Men Study (HIMS)
Source: BMC Geriatr. 2015 Dec 9;15:164. doi: 10.1186/s12877-015-0157-2 (PMC4674929; doi:10.1186/s12877-015-0157-2)
Supplement: Additional file 3: Table S3. — Incidence of major vascular events versus recreational physical activity, with progressive adjustment for potential confounders (among 7564 participants) (PDF 21 kb) [file 12877_2015_157_MOESM3_ESM.pdf]

Supplementary Table 3: Incidence of major vascular events versus recreational physical activity, with progressive adjustment for potential confounders (among 7564 participants)

Physical activity, MET-hours/week	Median MET-hours/week	Number of events	Adjusted for age, education and smoking	Plus place of birth, marital status, quantity of weekly alcohol intake, frequency that salt is added to food	Plus diabetes, use of blood pressure medication, use of cholesterol medication*
			HR (95% CI)	HR (95% CI)	HR (95% CI)
0	0.0	443	1.00 (0.91-1.10)	1.00 (0.91-1.10)	1.00 (0.91-1.10)
1-14	9.0	274	0.88 (0.79-1.00)	0.88 (0.78-1.00)	0.85 (0.75-0.96)
15-24	17.5	282	0.81 (0.72-0.91)	0.83 (0.74-0.94)	0.81 (0.72-0.91)
25-39	30.0	263	0.81 (0.72-0.91)	0.82 (0.73-0.93)	0.80 (0.71-0.91)
≥40	54.0	295	0.80 (0.71-0.89)	0.80 (0.71-0.90)	0.80 (0.71-0.91)

HR=hazard ratio.
* n.b. These risk factors may have role in mediating the effect of physical activity on risk of major vascular events.
