# Supplementary material for: Physical activity and vascular disease in a prospective cohort study of older men: The Health In Men Study (HIMS)
Source: BMC Geriatr. 2015 Dec 9;15:164. doi: 10.1186/s12877-015-0157-2 (PMC4674929; doi:10.1186/s12877-015-0157-2)
Supplement: Additional file 4: Table S4. — Excluding first 2 years of follow-up: hazard ratios for incidence of major vascular events in active versus inactive men, by age at risk (among 7350 participants) (PDF 14 kb) [file 12877_2015_157_MOESM4_ESM.pdf]

Supplementary Table 4: Excluding first two years of follow-up: hazard ratios for incidence of major vascular events in active versus inactive men, by age at risk (among 7350 participants)

	Number of events	Mean age at event, years	Hazard ratio (95% CI)
**Age at risk, years**			
65-74	199	72.5	0.89 (0.69-1.17)
75-84	840	70.0	0.80 (0.61-1.04)
85-94	305	88.0	0.66 (0.51-0.84)
			Trend, 3 groups: χ^2_1=2.7 (P=0.10)
**Overall**	1344	80.7	0.81 (0.76-0.86)

Hazard ratios (adjusted for age at risk, education and smoking) for incidence of major vascular events in participants reporting some physical activity versus those reporting no recreational physical activity (referent), by age at risk.
