# Supplementary material for: Physical activity and vascular disease in a prospective cohort study of older men: The Health In Men Study (HIMS)
Source: BMC Geriatr. 2015 Dec 9;15:164. doi: 10.1186/s12877-015-0157-2 (PMC4674929; doi:10.1186/s12877-015-0157-2)
Supplement: Additional file 5: Table S5. — Hazard ratios for cause-specific incidence of major vascular events versus recreational physical activity, with further adjustment for BMI (among 7564 participants) (PDF 33 kb) [file 12877_2015_157_MOESM5_ESM.pdf]

Supplementary Table 5: Hazard ratios for cause-specific incidence of major vascular events versus recreational physical activity, with further adjustment for BMI (among 7564 participants)

Physical activity, MET-hours per week	Median MET-hours per week	Ischaemic heart disease		Stroke		Other vascular		All major vascular events	
		n	Hazard ratio (95% CI)	n	Hazard ratio (95% CI)	n	Hazard ratio (95% CI)	n	Hazard ratio (95% CI)
0	0.0	243	1.00 (0.88-1.14)	147	1.00 (0.85-1.18)	53	1.00 (0.76-1.32)	443	1.00 (0.91-1.10)
1-14	9.0	128	0.77 (0.65-0.91)	112	1.10 (0.91-1.32)	34	0.93 (0.67-1.31)	274	0.90 (0.80-1.01)
15-24	17.5	153	0.82 (0.70-0.96)	99	0.87 (0.71-1.06)	30	0.77 (0.54-1.10)	282	0.83 (0.74-0.93)
25-39	30.0	150	0.87 (0.74-1.02)	87	0.83 (0.68-1.03)	26	0.70 (0.48-1.03)	263	0.84 (0.74-0.95)
≥40	54.0	159	0.82 (0.70-0.95)	106	0.92 (0.76-1.11)	30	0.71 (0.49-1.01)	295	0.84 (0.75-0.94)
		Trend, 5 groups: χ^2_1=1.9 (P=0.17)		Trend, 5 groups: χ^2_1=1.4 (P=0.23)		Trend, 5 groups: χ^2_1=3.0 (P=0.09)		Trend, 5 groups: χ^2_1=5.1 (P=0.02)	

Hazard ratios adjusted for age at risk, education, smoking and BMI. In the lower physical activity range (<25 MET-hours per week), 10 MET-hours per week greater physical activity was associated with 10% lower risk of all major vascular events combined (hazard ratio 0.90 [95% CI 0.83-0.97], P=0.007), adjusting for age at risk, education, smoking and BMI.
